# Increased reliance on coronary perfusion for cardiorespiratory performance in seawater-acclimated rainbow trout

**DOI:** 10.1242/jeb.244733

**Published:** 2023-02-17

**Authors:** Nicklas Wallbom, Lucas A. Zena, Tristan J. McArley, Andreas Ekström, Michael Axelsson, Albin Gräns, Erik Sandblom, Daniel Morgenroth

**Affiliations:** ^1^ Department of Biological and Environmental Sciences, University of Gothenburg, 405 30 Gothenburg, Sweden; ^2^ Department of Animal Environment and Health, Swedish University of Agricultural Sciences, 405 30 Gothenburg, Sweden

**Keywords:** Physiology, Exercise, Coronary circulation, Salinity

## Abstract

Salmonid ventricles are composed of spongy and compact myocardium, the latter being perfused via a coronary circulation. Rainbow trout (*Oncorhynchus mykiss*) acclimated to sea water have higher proportions of compact myocardium and display stroke volume-mediated elevations in resting cardiac output relative to freshwater-acclimated trout, probably to meet the higher metabolic needs of osmoregulatory functions. Here, we tested the hypothesis that cardiorespiratory performance of rainbow trout in sea water is more dependent on coronary perfusion by assessing the effects of coronary ligation on cardiorespiratory function in resting and exhaustively exercised trout acclimated to fresh water or sea water. While ligation only had minor effects on resting cardiorespiratory function across salinities, cardiac function after chasing to exhaustion was impaired, presumably as a consequence of atrioventricular block. Ligation reduced maximum O_2_ consumption rate by 33% and 17% in fish acclimated to sea water and fresh water, respectively, which caused corresponding 41% and 17% reductions in aerobic scope. This was partly explained by different effects on cardiac performance, as maximum stroke volume was only significantly impaired by ligation in sea water, resulting in 38% lower maximum cardiac output in seawater compared with 28% in fresh water. The more pronounced effect on respiratory performance in sea water was presumably also explained by lower blood O_2_ carrying capacity, with ligated seawater-acclimated trout having 16% and 17% lower haemoglobin concentration and haematocrit, respectively, relative to ligated freshwater trout. In conclusion, we show that the coronary circulation allows seawater-acclimated trout to maintain aerobic scope at a level comparable to that in fresh water.

## Teaser

**Summary:** Increased myocardial compaction in seawater-acclimated rainbow trout is associated with greater reliance on coronary perfusion for cardiorespiratory performance.

## INTRODUCTION

The osmoregulatory mechanisms utilized by euryhaline fishes vary depending on the surrounding environmental salinity. In the hypo-osmotic freshwater environment, fish compensate for the passive loss of ions and gain of water across the epithelia via dilute urine production and active branchial ion absorption (Perry et al., 2003). Conversely, sea water is hyperosmotic compared with the body fluids of teleosts, which leads to a passive gain of ions and loss of water (Laverty and Skadhauge, 2012). Thus, salmonids (e.g. rainbow trout, *Oncorhynchus mykiss*) transitioning from fresh water to sea water undergo a series of behavioural and physiological changes to cope with the reversal of the osmotic gradient. As salinity increases, they immediately start drinking water to avoid dehydration (Kirsch and Meister, 1982; Smith, 1930; Grosell, 2011; Brijs et al., 2015). The imbibed water is processed in the gastrointestinal tract and passively absorbed in the intestine coupled to the trans-epithelial osmotic gradient produced by enterocytic Na^+^/K^+^-ATPases (Grosell, 2006). In seawater-acclimated rainbow trout, this intestinal water absorption is associated with a twofold increase in gastrointestinal blood flow, that aids the absorption of water and ions (Brijs et al., 2016). The elevated gastrointestinal blood flow is driven by stroke volume (SV)-mediated elevations of cardiac output (CO), while heart rate (*f*_H_) is unchanged, and an increased proportion of CO is directed to the gastrointestinal tract, probably through regional changes in vascular resistance (Maxime et al., 1991; Brijs et al., 2015, 2016; Sundell et al., 2018; Morgenroth et al., 2019).

A rise in CO (e.g. during exercise and warming) can also increase the transport of O_2_ from the gills to the peripheral tissues and influence metabolic rate (*Ṁ*_O_2__; Ekström et al., 2017, 2018). The standard metabolic rate (SMR) of adult rainbow trout acclimated to sea water and fresh water is similar despite the potential differences in energetic costs associated with acclimating to different salinities (Brijs et al., 2015). Still, any constraints to maximum CO and/or tissue oxygen extraction (i.e. the arteriovenous oxygen content difference; A–VO_2_) during exercise at different salinities may limit the maximum metabolic rate (MMR) and reduce aerobic scope (AS). Such reductions could have ecological consequences for wild salmonids, as AS represents the energy available for aerobic performance and is associated with migratory and reproductive success (e.g. sockeye salmon, *Oncorhynchus nerka*: Pörtner et al., 2017; Eliason et al., 2011). While it is still unclear how MMR is affected by sea water acclimation in rainbow trout, MMR in wild sockeye salmon returning from the sea is similar in sea water and after re-acclimation to fresh water (Wagner et al., 2006). Additionally, it remains unknown whether maximum CO differs between seawater- and freshwater-acclimated trout. However, if maximum CO were similar across salinities, CO scope (i.e. the difference between maximum and resting CO) will be lower in sea water because of the higher resting CO, thus reducing AS unless compensatory increases in A–VO_2_ occur.

The ventricle of most fishes is entirely composed of spongy myocardium, which receives O_2_ from the poorly oxygenated venous blood that fills the lumen between heart beats (Bone et al., 1995; Farrell, 2011; Farrell and Pieperhoff, 2011). However, about one-third of all teleosts, including salmonids, possess a coronary circulation, which supplies the outer compact ventricular myocardium with oxygenated arterial blood from the gills (Axelsson, 1995; Farrell et al., 2012). Coronary blood flow supports cardiac performance and increases when cardiac O_2_ requirements increase (Gamperl et al., 1995; Ekström et al., 2017). When coronary blood flow is impaired by, for example, coronary arteriosclerosis, which is a fact of life for wild and farmed salmonids, cardiac performance may thus be compromised (Farrell, 2002; Brijs et al., 2020). This can be studied by surgically abolishing coronary blood flow by coronary ligation. Coronary ligation reduces SV in freshwater-acclimated rainbow trout, while CO is maintained by compensatory elevations in *f*_H_ (Gamperl et al., 1994; Ekström et al., 2017, 2018, 2019; Morgenroth et al., 2021; Zena et al., 2021). However, the ligation results in a reduced maximum CO (Ekström et al., 2019), and *in vitro* studies indicate this is due to loss of cardiac contractility (Agnisola et al., 2003). Consistent with these effects on cardiac performance, SMR is unaffected by coronary ligation, whereas post-exercise MMR is reduced and associated with a 29% drop in AS (Ekström et al., 2018). Not surprisingly, a blockade of coronary perfusion to the heart also reduces the critical swimming speed (Farrell and Steffensen, 1987), as well as acute temperature and hypoxia tolerance in trout (Morgenroth et al., 2021; Ekström et al., 2017, 2019). Even so, the role of the coronary circulation at different salinities in euryhaline fishes is largely unexplored. For example, no one has examined the influence of coronary blood flow on maximal cardiorespiratory performance or recovery from exercise at different salinities.

A recent study demonstrated that seawater-acclimated rainbow trout had more compact myocardium compared with that of conspecifics in fresh water, suggesting an increased dependency on the coronary circulation to maintain cardiac and aerobic performance in sea water (Brijs et al., 2017a). To address this possibility, we examined the effects of coronary ligation on cardiorespiratory performance in groups of rainbow trout acclimated to fresh water or sea water. We hypothesized that rainbow trout rely more on coronary blood flow when at sea versus in fresh water and that this reliance is most pronounced during periods of physical activity. Consequently, we expected that the proportion of ventricular compact myocardium (i.e. relative compact mass) would be higher in seawater-acclimated fish, and that maximum CO, MMR and AS would be constrained in coronary-ligated rainbow trout. In addition, fish acclimated to sea water were expected to suffer larger constraints compared with trout acclimated to fresh water. To test this hypothesis, we measured relative compact mass together with cardiac (CO, *f*_H_, and SV) and respiratory (*Ṁ*_O_2__) performance in coronary-ligated or sham-operated rainbow trout acclimated to fresh water or sea water, before and after an exhaustive exercise protocol. We thus measured resting, maximal and scope for CO, *f*_H_ and SV alongside SMR, MMR and AS. In addition, we measured the cardiorespiratory dynamics after exercise to evaluate the ability of the fish to recover from physical activity. This included measurements of excess post-exercise oxygen consumption (EPOC), which represents the O_2_ required to replenish O_2_ stores, restore the balance of biomolecules such as phosphates, lactate and glycogen, and re-establish acid–base and osmotic balance following exercise (Gaesser and Brooks, 1984; Scarabello et al., 1992; Kieffer, 2000). Finally, to assess blood oxygen transporting capacity of the different groups, haemoglobin concentration ([Hb]) and haematocrit (Hct) were measured.

## MATERIALS AND METHODS

### Fish and holding conditions

Sexually immature rainbow trout of mixed sex (see Table 1 for biometric details) were obtained from a commercial fish farm (Vänneåns fiskodling AB, Knäred, Sweden) and held in fresh water for at least 4 weeks to allow acclimation to laboratory conditions. Half of the fish (*n*=17; see Table 1) were then acclimated to sea water (salinity 31–34 ppt; sea salt from Aquaforest, Brzesko, Poland) for at least 4 additional weeks prior to the experiment, while the rest of the fish remained in fresh water. All fish were held in 2000 l tanks continuously supplied with recirculating aerated water at their respective salinity, maintained at ∼11°C under a 12 h:12 h light:dark photoperiod. Fish were fed twice per week with commercial pellets (7 mm, Protec Trout pellets, Skretting, Stavanger, Norway), and were fasted for 3 days prior to the experiments. The study was covered by ethical permits 165-2015 and 5.8.18-10907/2020, approved by the regional ethical committee in Gothenburg.

**Table 1.**
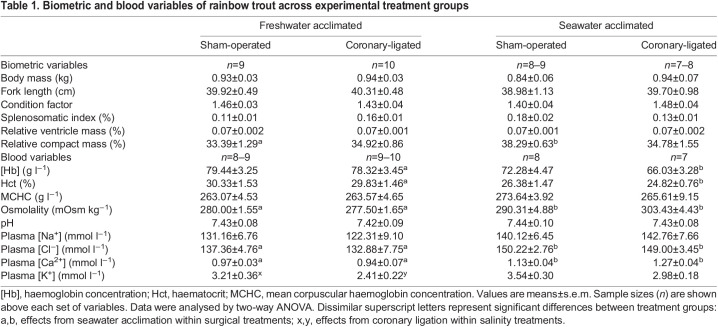
Biometric and blood variables of rainbow trout across experimental treatment groups

### Surgical instrumentation

Before surgery, all fish were anaesthetized in fresh water containing 150 mg l^−1^ MS-222 (ethyl-3-aminobenzoate methanesulphonic acid; Sigma Aldrich Inc., St Louis, MO, USA) buffered with 300 mg l^−1^ NaHCO_3_. When opercular movements ceased, body mass and fork length were measured before placing the fish laterally on its left side on a surgical table covered with wet foam. During surgery, a continuous flow of water at ∼10°C with a maintenance dose of MS-222 (75 mg l^−1^; buffered with 150 mg l^−1^ NaHCO_3_ in freshwater fish) was used to irrigate the gills. The ventral aorta and the coronary artery were exposed via an incision in the isthmus inside the opercular cavity and dissected free using blunt dissection to avoid damaging surrounding vessels and nerves, or puncturing the pericardium. At each salinity, one group of fish was coronary ligated, and another was sham operated. In coronary-ligated fish, blood flow through the coronary artery was permanently interrupted by tying a 6–0 silk suture around the vessel. The sham-operated fish underwent an identical surgical procedure, although the coronary artery was not ligated. Next, a 2.5 mm transit-time blood flow probe (L type, Transonic Systems, Ithaca, NY, USA) was placed around the ventral aorta. The probe was secured to the skin with two silk sutures, one inside the opercular cavity and one outside the opercular cavity. The lead from the flow probe was additionally secured in the skin in front of the dorsal fin with a single suture.

### Experimental protocol

Following surgery, the fish were transferred into custom-built 10 l PVC respirometers placed inside 230 l holding tanks supplied with 10°C aerated water. Recordings of *Ṁ*_O_2__, CO and *f*_H_ started immediately (see below for details). The fish were allowed to recover from surgery for ≥24 h before the start of the experimental protocol, after which SMR was determined for ≤6 h (see ‘Data acquisition and analytical approaches’, below). Thirty hours after surgery, the fish were removed from the respirometer into a net and transferred into a circular 100 l tank with running water at the appropriate salinity, where individual fish were manually chased for 5 min or until exhaustion (i.e. until the fish lost equilibrium; no significant differences). After the chase, the fish were immediately transferred back into the respirometers and recordings of CO, *f*_H_ and *Ṁ*_O_2__ were resumed and continued for at least 15 h. The fish were then euthanized by a sharp cranial blow and a ∼1 ml blood sample was immediately drawn from the caudal vessels for immediate determination of blood pH, Hct and [Hb]. The remaining blood was centrifuged at 10,000 ***g*** for 5 min and plasma samples were stored at −80°C for later analysis (see below). The spleen and heart ventricle were then dissected out, blotted, and weighed to determine their wet mass. The ventricle was preserved in 70% ethanol and stored in a fridge for further analysis of ventricular composition (see below).

### Data acquisition and analytical approaches

#### Biometric measurements

Condition factor was calculated as 100×body mass/fork length^3^ with body mass in grams and fork length in centimetres. Splenosomatic index was calculated as spleen mass/body mass×100. Relative ventricle mass was calculated as wet ventricular mass/body mass×100. The compact and spongy myocardium were separated and subsequently dried for at least 20 h at 60°C before determining their dry mass, following the protocol by Farrell et al. (2007). The proportion of ventricular compact myocardium (i.e. relative compact mass) was calculated as dry compact myocardial mass/dry ventricular mass×100.

#### Cardiorespiratory measurements

*Ṁ*_O_2__ was measured using automated intermittent flow respirometry. Briefly, air saturation in the respirometer was measured with an O_2_-optode connected to a FireSting O_2_ system (PyroScience, Aachen, Germany). The O_2_-optode was one-point calibrated, using 100% air-saturated water as reference. *Ṁ*_O_2__ was measured in automated cycles consisting of a flush time (inflow of oxygenated water into the respirometer) of 3–4 min and a measuring time (no inflow of oxygenated water) of 5–10 min, typically with shorter cycles just after the chase. The cycles were monitored and adjusted as needed, with the goal of maintaining air saturation in the respirometer above 80% at the end of each *Ṁ*_O_2__ cycle. Measurements of background microbial respiration were performed at the end of each experimental series following the removal of the fish, and the respirometers were then cleaned before housing another animal. The slope of the decline in air saturation when the flush pump was off was used to calculate *Ṁ*_O_2__ after correction for microbial background respiration according to Svendsen et al. (2016), as:
(1)

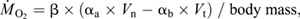
where β is the O_2_ solubility at a given salinity and temperature, α_a_ and α_b_ are the fish and background O_2_-consumption rates, respectively, calculated from the slope of the decline in percentage air saturation per second, *V*_t_ is the total volume of the respirometer and *V*_n_ is the net volume of the respirometer with a fish present (*V*_t_−body mass, where body mass is assumed to equal fish volume). The first 30–60 s of every measurement cycle were excluded to prevent including sections of non-linear decline in air saturation. Additionally, a coefficient of determination (*R*^2^) was computed for each slope, and slopes were only accepted when *R*^2^>0.90. SMR was defined as the mean of the lowest 20% of *Ṁ*_O_2__ measurements after recovery from surgery until the exercise protocol, excluding any outliers (i.e. mean±2 s.d.), and MMR was defined as the highest *Ṁ*_O_2__ measurement after exhaustive exercise (Clark et al., 2013).

Excess post-exercise oxygen consumption (EPOC) was calculated by trapezoidal integration of the area between the routine *Ṁ*_O_2__ values and the SMR+10% after exercise until recovery (Zhang et al., 2018; Lee et al., 2003). Recovery time (i.e. EPOC duration) was defined as the time after exercise when SMR+10% was reached (Zhang et al., 2018). The *Ṁ*_O_2__ data were smoothed to exclude elevations of *Ṁ*_O_2__ that were probably due to spontaneous activity. In brief, if *Ṁ*_O_2__ increased by at least 5% between two subsequent data points, that point was set equal to the previous point, and kept at that value until reaching a value within 5% of the starting value (Zhang et al., 2018). If a fish did not reach SMR+10% during recovery, it was excluded from further EPOC analyses. Because of the cardiorespiratory impairment following exercise in the ligated groups, the data were not smoothed during the first 2 h in these individuals.

All signals from the recording equipment were connected to a PowerLab system (ADInstruments, Sydney, NSW, Australia). The signals were recorded and analysed using LabChart Pro data acquisition software (v7.3.8; ADInstruments) at a sampling rate of 10 Hz. The signal from the flow probes was relayed to blood flow meters (models T402 and T403, Transonic Systems). Each flow probe was bench calibrated at ∼10°C according to the instructions in the user manual. In brief, the flow probe was placed around a calibration tubing in a water bath at 10°C. Water was pumped through the tubing using known flow rates (from 1 to 70 ml min^−1^) from a pulsatile pump (model 1407, Harvard Apparatus, Holliston, MA, USA) for 1 min per flow setting. The water was collected and weighed to determine the flow rate gravimetrically and plotted against the recorded flow to correct the *in vivo* measurement of CO. CO was determined as the mean blood flow during *Ṁ*_O_2__ measurements. Resting cardiorespiratory variables were defined as the means of measurements during SMR and maximum cardiorespiratory variables were defined as the value obtained at MMR. *f*_H_ was obtained during these periods as the frequency of pulses in the blood flow traces. SV was calculated as CO divided by *f*_H_.

#### Haematological measurements

We measured haemoglobin concentration ([Hb]) using a handheld Hb 201^+^ analyser (Hemocue, Ängelholm, Sweden), and corrected the values for fish blood according to Clark et al. (2008). Hct was determined by measuring the fraction of red blood cells after spinning the blood in microhaematocrit tubes at 10,000 ***g*** for 5 min. Mean corpuscular haemoglobin concentration (MCHC) was calculated as [Hb] divided by Hct. Plasma osmolality was determined from duplicate samples using a micro-osmometer (model 3320, Advanced Instruments, Norwood, MA, USA). Similarly, the plasma ion concentrations ([K^+^], [Ca^2+^], [Na^+^] and [Cl^−^]) were determined from duplicate samples using an electrolyte analyser (Convergys^®^ ISE Comfort, Convergent Technologies, Coelbe, Germany).

### Analytical and statistical approaches

Data exploration, calculations and statistical analyses were performed using R (v4.0.3; http://www.R-project.org/) with the car, emmeans and rstatix packages (Fox and Weisberg, 2019; https://CRAN.R-project.org/package=car; https://CRAN.R-project.org/package=emmeans; https://CRAN.R-project.org/package=rstatix). Final visualization was performed using Prism (v9.0.2; GraphPad Software, San Diego, CA, USA). Resting, maximum and scope of cardiorespiratory variables, as well as biometric and blood variables, were analysed by two-way analysis of variance (ANOVA). Whenever significant effects were found for either predictor (i.e. seawater acclimation or coronary ligation), the simple main effect of that predictor was explored at each level of the other predictor using Bonferroni correction of confidence intervals. To analyse cardiorespiratory dynamics over time after exercise, data were collected for each variable at 0, 10, 20, 30, 60, 120, 300, 600 and 900 min after exercise (or the temporally closest available data point) in each fish and analysed using mixed effects model ANOVA. Individual fish were included as subject variable, surgical treatment and salinity treatment as between-subject factors, and time as within-subject factor. The interaction terms between all factors were also included in the models. Whenever significant effects were found from either predictor (i.e. seawater acclimation or coronary ligation), the simple main effect of that predictor was explored at each time point using Bonferroni correction of confidence intervals.

The assumptions of all models were assessed by visual inspection of residual plots and formal residual analysis. Normality, homoscedasticity, homogeneity of covariances and extreme outliers were assessed with Shapiro–Wilk test, Levene's test, Box's *M*-test, and the box plot method, respectively. Sphericity was assessed using Mauchly's test, and the Greenhouse–Geisser sphericity correction was applied as needed. Extreme outliers were removed, and data were transformed as necessary to meet model assumptions. Resting CO and SV scope were not normally distributed and were log-transformed. To equalize variances, maximum *f*_H_ was square root transformed, and maximum SV was squared. Data from anaemic fish (Hct<20) were discarded. Significance was accepted when *P*<0.05. All data are presented as means±s.e.m.

## RESULTS

### Biometric characteristics of the experimental treatment groups

There were no differences in body mass, fork length, condition factor, splenosomatic index or relative ventricle mass across experimental treatment groups (Table 1). However, acclimation to sea water resulted in an increased proportion of ventricular compact myocardium in the sham-operated group (*F*_1,32_=9.93, *P*=0.004; Table 1).

### Effects of seawater acclimation on cardiorespiratory performance in sham-operated fish

**Fig. 1.**
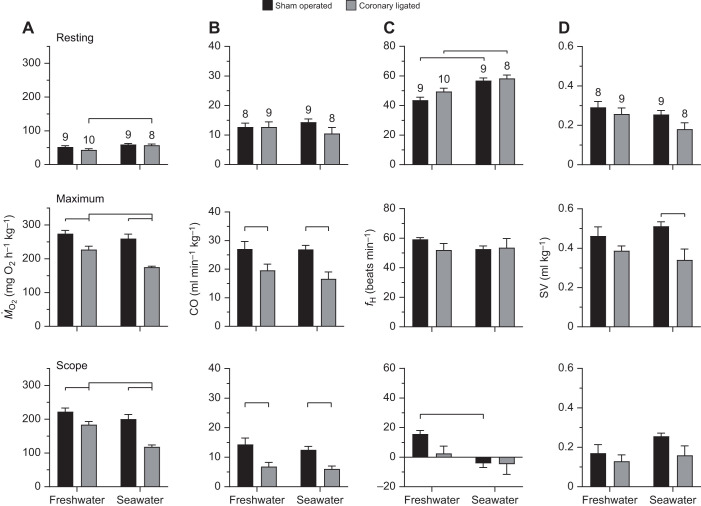
**Effects of seawater acclimation and coronary ligation on resting and maximum cardiorespiratory variables in rainbow trout.** Bar plots of resting (upper panels), maximum (middle panels) and scope (lower panels) of cardiorespiratory variables for (A) oxygen consumption rate (metabolic rate, *Ṁ*_O_2__), (B) cardiac output (CO), (C) heart rate (*f*_H_) and (D) stroke volume (SV) in sham-operated and coronary-ligated rainbow trout. Values are means±s.e.m., and numbers above bars in the upper panel denote sample sizes. Data were analysed by two-way ANOVA. Brackets denote significant (*P*<0.05) differences between groups and Bonferroni correction of confidence intervals was applied when simple main effects of either predictor were evaluated.

Seawater acclimation of sham-operated fish did not significantly affect SMR, MMR or AS (Fig. 1A). However, the resting *f*_H_ was higher in sea water (44±2 versus 57±2 beats min^−1^ in fresh water and sea water, respectively; *F*_1,32_=19.54, *P*<0.001), which rendered the *f*_H_ scope lower in sea water (16±2 versus −4±3 beats min^−1^ in fresh water and sea water, respectively; *F*_1,32_=9.57, *P*=0.004; Fig. 1C). Thus, the *f*_H_ scope was negative in seawater-acclimated trout because the maximum *f*_H_ after chasing stress was 5 beats min^−1^ lower than the resting *f*_H_ (Fig. 1C).

**Fig. 2.**
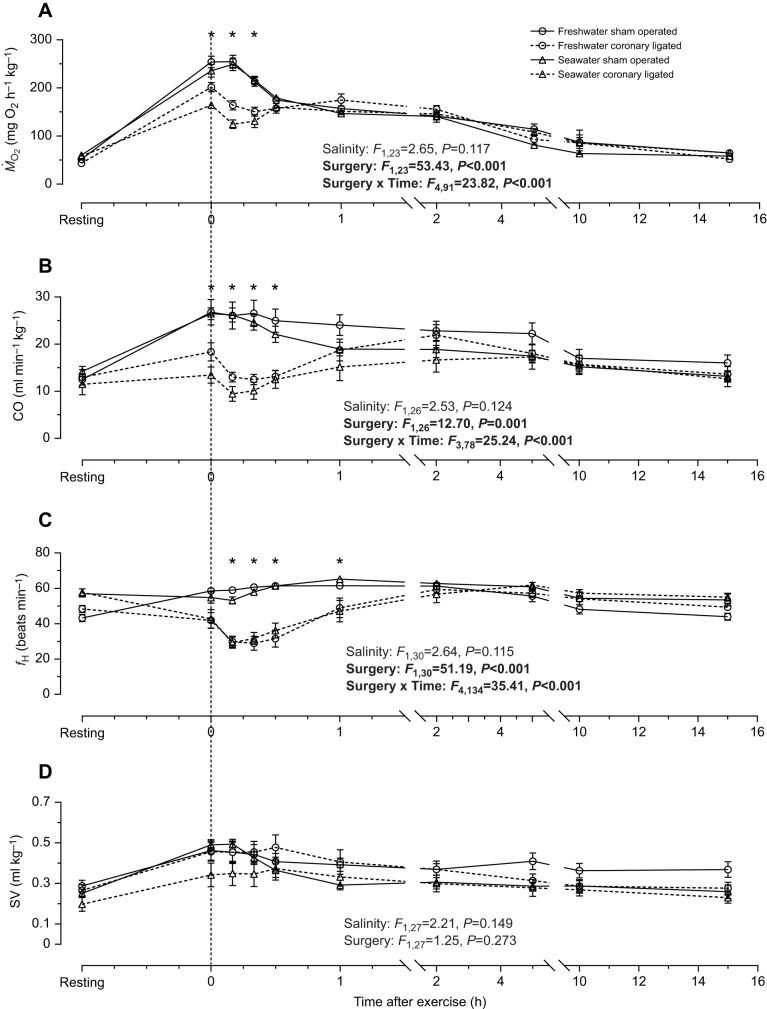
**Effects of seawater acclimation and coronary ligation on post-exhaustive cardiorespiratory dynamics.** (A) *Ṁ*_O_2__, (B) CO, (C) *f*_H_ and (D) SV over time after exhaustive exercise for sham-operated and coronary-ligated fish. Data are means±s.e.m. The vertical dashed line indicates the time of exhaustive exercise. Data were analysed by mixed effects model ANOVA on the same sample sizes as in Fig. 3 and the statistical output is shown in each panel. Asterisks denote time points at which significant (*P*<0.05) effects from coronary ligation were found and Bonferroni correction of confidence intervals was applied when simple main effects of coronary ligation were evaluated.

**Fig. 3.**
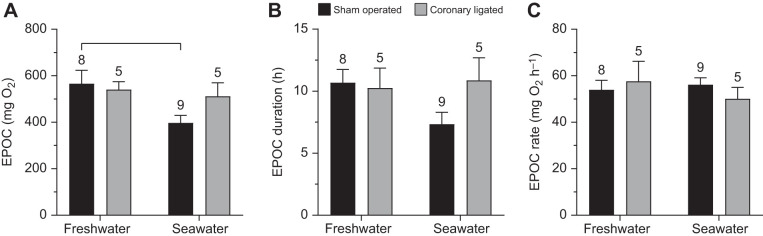
**Effects of seawater acclimation and coronary ligation on excess post-exercise oxygen consumption.** (A) Excess post-exercise oxygen consumption (EPOC), (B) EPOC duration and (C) EPOC rate in sham-operated and coronary-ligated rainbow trout. Data are means+s.e.m. Numbers above error bars indicate sample sizes. Data were analysed by two-way ANOVA. Brackets denote significant differences (*P*<0.05) between groups and Bonferroni correction of confidence intervals was applied when simple main effects of either predictor were evaluated.

There were no apparent effects of seawater acclimation on post-exhaustive cardiorespiratory dynamics (Fig. 2). However, the overall EPOC was significantly lower in sea water (608.0±65.7 versus 397.3±32.0 mg kg^−1^ in fresh water and sea water, respectively; *F*_1,23_=6.07, *P*=0.022; Fig. 3A), although the duration and rate of EPOC repayment were not affected (Fig. 3B,C).

### Effects of coronary ligation on cardiorespiratory performance across salinities

Within salinity treatment groups, coronary ligation did not have any significant effects on resting cardiorespiratory function (Fig. 1). However, SMR in coronary-ligated trout was significantly higher in sea water than in fresh water (57.0±4.0 versus 43.2±3.8 mg O_2_ h^−1^ kg^−1^; *F*_1,32_=6.60, *P*=0.015; Fig. 1A). No such effects were found for resting CO or SV, although as in sham-operated fish, the resting *f*_H_ was higher in sea water relative to fresh water (50±2 versus 58±2 beats min^−1^; *F*_1,32_=8.50, *P*=0.007; Fig. 1C).

Coronary ligation had a clear negative effect on MMR and AS in both fresh water and sea water. In fresh water, MMR was significantly lower in ligated fish (274.3±9.61 versus 227.2±10.2 mg O_2_ h^−1^ kg^−1^; *F*_1,32_=11.80, *P*=0.002), and so was AS (222.4±10.5 versus 184.0±9.4 mg O_2_ h^−1^ kg^−1^; *F*_1,32_=7.18, *P*=0.012; Fig. 1A). Similarly, in sea water, MMR was lower in ligated fish (259.9±13.0 versus 175.3±2.8 mg O_2_ h^−1^ kg^−1^; *F*_1,32_=33.96, *P*<0.001) and so was AS (200.6±13.7 versus 118.3±5.4 mg O_2_ h^−1^ kg^−1^; *F*_1,32_=29.46, *P*<0.001; Fig. 1A). Importantly, the differences in MMR and AS in ligated relative to sham-operated trout were 79% and 114% larger in sea water than in fresh water (*F*_1,32_=13.41, *P*<0.001 and *F*_1,32_=19.68, *P*<0.001, respectively; Fig. 1A). Even so, the effect of coronary ligation on maximum CO was similar across salinities, resulting in consistently lower maximum CO in fresh water (27.1±2.5 versus 19.6±2.0 ml min^−1^ kg^−1^) and sea water (26.9±1.4 versus 16.6±2.4 ml min^−1^ kg^−1^; *F*_1,30_=11.37, *P*=0.002; Fig. 1B). The effect on CO scope was also similar across salinities (14.4±2.0 versus 6.9±1.4 ml min^−1^ kg^−1^; *F*_1,30_=12.90, *P*=0.001 in fresh water, and 12.5±1.2 versus 6.1±0.9 ml min^−1^ kg^−1^; *F*_1,30_=12.90, *P*=0.001 in sea water; Fig. 1B). The lower maximum CO in coronary-ligated fish was explained by a lower maximum SV, as maximum *f*_H_ was similar across treatment groups (Fig. 1C,D). Yet, this effect was only significant in sea water (0.51±0.02 versus 0.34±0.06 ml kg^−1^; *F*_1,30_=8.29, *P*=0.007; Fig. 1D). Accordingly, coronary ligation did not affect the *f*_H_ scope when compared across salinities, although it should be noted that, as for sham-operated fish, *f*_H_ scope was negative in ligated fish in sea water (Fig. 1C).

Post-exhaustive *Ṁ*_O_2__ and CO dynamics were markedly affected by coronary ligation, as both variables immediately decreased after chase in coronary-ligated fish and remained lower than their sham-operated counterparts for at least 30 min at both salinities (Fig. 2A,B). The reduction of *Ṁ*_O_2__ and CO was mainly driven by a bradycardia at both salinities, starting a few minutes after the chase and persisting for approximately 30 min before slowly returning to levels comparable to those of sham-operated fish over the course of an hour (Fig. 2C). Despite the effects of coronary ligation on post-chase cardiorespiratory dynamics, EPOC, rate of EPOC repayment and EPOC duration were not affected by coronary ligation (Fig. 3A,B). An important caveat, however, is that EPOC could not be calculated for roughly half of the coronary-ligated treatment group as they never reached a point of recovery throughout the pre-set length of the experimental protocol.

### Haematological responses to seawater acclimation and coronary ligation

Plasma osmolality was higher in seawater-acclimated fish in both the sham-operated and coronary-ligated groups (*F*_1,30_=4.73, *P*=0.038 and *F*_1,30_=29.08, *P*<0.001, respectively; Table 1). This difference was mainly due to higher plasma [Cl^−^] in both sham-operated and coronary-ligated treatments (*F*_1,28_=15.55, *P*<0.001 and *F*_1,28_=4.21, *P*=0.049, respectively; Table 1). Plasma [K^+^] was lower in coronary-ligated fish in fresh water (*F*_1,30_=4.43, *P*=0.044; Table 1). Furthermore, acclimation to sea water was associated with lower [Hb] and Hct, but only in the coronary-ligated group (*F*_1,30_=5.18, *P*=0.030 and *F*_1,30_=5.91, *P*=0.021, respectively; Table 1).

## DISCUSSION

### Coronary blood flow has added importance for maximal cardiorespiratory performance in sea water

This is the first study comparing the role of the coronary circulation for cardiorespiratory performance in salmonid fish at different salinities. We show that complete blockade of coronary blood flow to the heart by surgical ligation had minor effects in resting conditions, but dramatic effects on maximum cardiorespiratory performance. These effects were salinity dependent; while MMR was 17% lower in coronary-ligated fish relative to sham-operated fish in fresh water, the effect was significant and roughly twice as high (33% lower MMR) in sea water. Consequently, AS after ligation was 17% lower in fresh water and 41% lower in sea water. The over twofold larger effect of ligation on respiratory performance in sea water clearly indicates an increased reliance on coronary perfusion to sustain maximum cardiorespiratory performance in sea water. Corroborating this conclusion, the exhaustive exercise protocol used here typically caused an immediate bradycardia in ligated trout, resulting in low *f*_H_ scopes that were even negative in sea water. In addition, although maximum CO did not differ significantly between coronary-ligated fish across salinity treatments, the maximum SV of ligated fish was significantly lower than that of sham-operated fish in sea water, while ligation had no significant effect on maximum SV in fresh water. As a result, the maximum CO in ligated fish was 38% lower in sea water, versus 28% lower in fresh water. This points to a larger cardiac impairment from ligation in seawater-acclimated trout. Still, it should be noted that the lower maximum CO in coronary-ligated fish does not fully explain the marked difference in maximum respiratory performance across salinities. These differences were probably also due to lower blood O_2_ carrying capacity in seawater trout, as indicated by the 16% lower [Hb] and 17% lower Hct in ligated fish acclimated to sea water.

There is no established consensus about the haematological responses of fish to different environmental salinities. For example, in contrast to the current study, rearing rainbow trout and tambaqui (*Colossoma macropomum*) in brackish water has been observed to increase Hct and [Hb] when compared with fresh water (Hosseinzadeh Sahafi et al., 2013; Fiúza et al., 2015). Similarly, the Hct of rainbow trout was higher after 5 weeks at 18 ppt compared with that in fresh water (Morgan and Iwama, 1991). The same authors observed no effect in steelhead trout, and the reverse response was seen in Chinook salmon (*Oncorhynchus tshawytscha*), where Hct was lower after 56 days at >20 ppt, as in the present study (Morgan and Iwama, 1991). Also consistent with our study, Hct was lower in seawater-acclimated coho salmon (*Oncorhynchus kisutch*) after 60 and 150 days (Fang et al., 2019), and Brauner et al. (1992) showed in the same species that fish swimming in sea water displayed a reduced Hct, while the response was the opposite in fresh water. In the present study, only ligated trout exhibited lower Hct and [Hb] in sea water, suggesting that coronary ligation might exacerbate the reduction of [Hb] that can be associated with seawater acclimation (e.g. Morgenroth et al., 2019; Brijs et al., 2017a; Haney et al., 2003). In fact, even in fresh water, [Hb] has been observed to be lower in coronary-ligated fish following warming, and the elevations in [Hb] typically observed in fish exposed to warming are attenuated (Ekström et al., 2019; Morgenroth et al., 2021). Thus, it appears that coronary ligation in combination with environmental (e.g. temperature, salinity) changes may affect haematological parameters, although the underlying mechanisms remain unknown.

### Coronary ligation impairs cardiorespiratory function following exercise

**Fig. 4.**
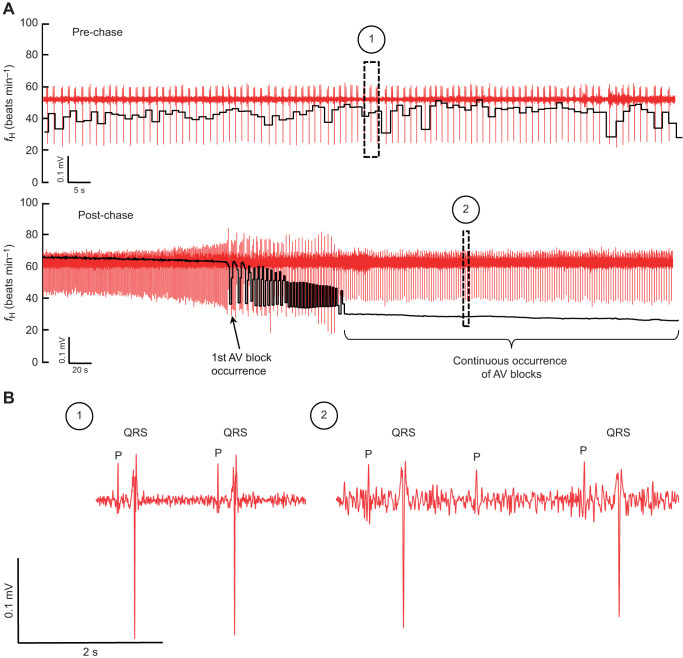
**Representative example of an electrocardiogram of coronary-ligated rainbow trout before and after exhaustive chasing.** Data were obtained in a previous study (Zena et al., 2021). (A) Electrocardiogram traces (red) from an extended recording overlayed with *f*_H_ calculated in LabChart (black) pre- and post-chasing. (B) Close-up view of individual P-waves and QRS-complexes before (1) and after (2) chasing (corresponding to the boxed regions in A). Before chasing occurred, every P-wave was followed by a QRS-complex, and *f*_H_ was around 40 beats min^−1^. After chasing, *f*_H_ initially increased, but within minutes a second-degree atrioventricular (AV) block (*P*-waves present, but missing QRS-complex) developed, which caused *f*_H_ to collapse.

Following exercise, the coronary-ligated trout experienced a transient reduction in *f*_H_, CO and routine *Ṁ*_O_2__. A previous study from our group has identified a first-degree atrioventricular (AV) block in resting coronary-ligated rainbow trout, which manifest as an increased duration of the P–R interval in the electrocardiogram (Brijs et al., 2020; see also Merideth and Pruitt, 1973). In a more recent study, the coronary artery of rainbow trout was ligated, and the fish were subsequently stressed using an identical chasing protocol to that in the present study (Zena et al., 2021). Re-analysis of data from that study suggests that the cardiac electrical conduction of the myocardium was impaired in some coronary-ligated trout chased after 3 days of recovery from coronary ligation. A representative example is shown in Fig. 4 where missing QRS-complexes after the chase period resulted in an approximate P-wave:QRS-complex ratio of 2:1, indicating the occurrence of a second-degree AV block. Based on this, we propose that the cardiorespiratory impairment after exercise in coronary-ligated trout results from AV block. Similarly, depression of *f*_H_ at critically high temperatures is also due to a failure of ventricular excitation and manifests as a second-degree AV block (Haverinen and Vornanen, 2020). The underlying mechanism may relate to an imbalance of Na^+^ and K^+^ flux rates across the myocardial cell membranes as a result of malfunction of voltage-gated ion channels, as seen at high temperature *in vitro* in salmonids (Vornanen, 2020). Indeed, plasma [K^+^] consistently lower in ligated freshwater trout in this study, which may have disrupted these ion flux rates. Plasma [K^+^] was also lower in sea water, although the effect was not significant. Furthermore, it is noteworthy that AV block may be elicited by exercise in humans as a consequence of myocardial ischaemia (i.e. coronary disease; Aizawa and Kawamura, 2021). As the AV canal in rainbow trout is composed of compact myocardium (Icardo and Colvee, 2010; Hassinen et al., 2021) and is therefore likely to be perfused by coronary blood, coronary ligation might have a similar effect and disrupt the maintenance of membrane potential by impairing O_2_ delivery and thus production of ATP necessary for Na^+^/K^+^-ATPases in the AV canal. While this needs to be confirmed experimentally, it can be noted that one of the sham-operated fish exhibited clear signs of coronary obstruction following exhaustive exercise (i.e. bradycardia), and upon dissection, the coronary artery was found to be severely swollen and had a dark colour, suggesting that the coronary artery was occluded, perhaps by arteriosclerosis or thrombosis. Those data were excluded from analyses, but indicate that the results obtained here are relevant to fish outside the laboratory, not least from a welfare perspective, as cardiac disease (e.g. coronary arteriosclerosis) is prevalent in both wild and farmed fish (Farrell, 2002; Brijs et al., 2020).

To the best of our knowledge, this is also the first study examining the importance of coronary perfusion on EPOC at different salinities. Here, we measured EPOC as the accumulated *Ṁ*_O_2__ in excess of SMR after exhaustive exercise until *Ṁ*_O_2__ had returned to SMR+10%. Notably, in 44% of the coronary-ligated trout, SMR+10% did not recover during the 15 h post-chase period, compared with 6% of the sham-operated trout. While we obviously could not determine the EPOC and its duration in these fish, this clearly suggests that at least the duration of EPOC was prolonged when coronary blood flow was abolished. Assuming that there were no differences in the routine energetic demands among experimental groups during chasing, the limitations on maximal cardiorespiratory performance with coronary ligation are expected to increase the reliance on anaerobic metabolism during acute chasing stress. The transient, yet severe, cardiorespiratory impairment in coronary-ligated fish discussed above represents a further limitation to recovery as O_2_ transport was compromised during this period. Together, this could prolong the metabolic imbalances due to a continued reliance on anaerobic metabolism after chasing, thus explaining the increased duration of EPOC. Even though our data do not support an increased EPOC in ligated trout, a prolonged EPOC duration has potential ecological consequences, as it may influence how often fish can engage in burst swimming to, for example, avoid predation or catch prey (Killen et al., 2015).

### Seawater acclimation is associated with increased ventricular compaction and a lower O_2_ debt

There were no differences in relative ventricle mass among experimental treatment groups, but consistent with previous findings, the relative mass of compact myocardium increased with seawater acclimation in the sham-operated group (Brijs et al., 2017a). This effect was not observed in the ligated group, and while there were no effects of ligation on the relative mass of compact myocardium in either salinity, ligated trout in seawater had a numerically lower relative mass of compact myocardium compared with sham-operated fish. Previously reported effects of ligation on the relative mass of compact myocardium in fresh water range from no effect (Morgenroth et al., 2021) to a significantly lower proportion of compact myocardium in ligated fish (Zena et al., 2021). While the underlying causes of these discrepancies elude us, our results suggest that seawater-acclimated fish have an increased reliance on coronary blood flow for the maintenance of compact myocardial mass and that ligation reverses the cardiac remodelling associated with seawater acclimation.

It remains unclear what mechanism triggers the cardiac remodelling in sea water but an increase in plasma cortisol could be a candidate. Indeed, freshwater rainbow trout fed a cortisol-spiked diet elevating plasma cortisol develop increased relative compact mass, although such feeding regimes also increase the overall relative ventricle mass which is generally considered to reflect pathological cardiac growth (Johansen et al., 2017). Nonetheless, plasma cortisol is elevated during natural and induced smoltification and triggers various branchial and gastrointestinal changes associated with osmoregulation (Björnsson et al., 2011). Thus, it is possible that elevated plasma cortisol might produce adaptive cardiac changes such as increased ventricular compaction, as observed here.

Surprisingly, neither resting SV nor CO was changed by seawater acclimation in the present study, while *f*_H_ increased. This pattern contrasts with several previous studies observing increases in resting CO and SV with a constant *f*_H_ in rainbow trout following seawater acclimation (Sundell et al., 2018; Brijs et al., 2016, 2017a). The reason for these discrepancies remains unclear. While the maximum and scope values for CO and SV were not affected by seawater acclimation either, the elevated resting *f*_H_ in sham-operated trout acclimated to sea water resulted in a reduced scope for *f*_H_. Taken together, the presented data suggest that maximum cardiac performance was maintained across salinities. Thus, our initial hypothesis that seawater acclimation elevates maximum CO can be rejected.

When comparing across salinities, our data indicate that the total EPOC and its duration were reduced in seawater-acclimated trout despite identical chasing protocols. This suggests that the anaerobic costs associated with exhaustive exercise are lower in sea water. Interestingly, the intestine of rainbow trout acclimating to sea water shows signs of improved mitochondrial coupling and increased lactate dehydrogenase activity, indicating an improved anaerobic capacity and an enhanced efficiency of ATP production (Brijs et al., 2017b). Such metabolic changes might lessen the energetic constraints on recovery from exercise and potentially result in lower EPOC, although this remains speculative. Independently of the mechanisms behind the lower EPOC in sea water, the difference in plasma osmolality between acclimation groups in the present study was subtle compared with previous work (Morgenroth et al., 2019; Brijs et al., 2017a), indicating a high osmoregulatory capacity in the seawater-acclimated fish in the present study that could have potentially contributed to reduce the osmotic imbalances imposed by chasing stress.

### Future perspectives and significance

Climate change is predicted to affect the frequency and severity of salinity variations (Kültz, 2015). This study provides further insight into the cardiorespiratory changes, including coronary O_2_ supply to the heart, that occur in euryhaline salmonids acclimating to sea water. We show that rainbow trout acclimated to sea water have a maximum cardiorespiratory performance that matches that of freshwater-acclimated conspecifics when the coronary circulation is intact. However, after complete blockade of coronary blood flow, the negative effects on cardiorespiratory performance were larger in sea water, indicating increased reliance on coronary perfusion. Moreover, our data indicate that the cardiac electrical conduction is impaired in coronary-ligated trout, which manifests as post-chase bradycardia and a general cardiorespiratory impairment regardless of salinity. These results thus provide further evidence of the adaptive significance of the coronary circulation in enhancing aerobic performance of some fishes (e.g. migratory salmonids), particularly in species with a euryhaline life history.
